# Supplementary material for: The hiuABC operon mediates xenosiderophore utilization in Caulobacter crescentus
Source: J Bacteriol. 2026 Feb 4;208(3):e00400-25. doi: 10.1128/jb.00400-25 (PMC13001220; doi:10.1128/jb.00400-25)
Supplement: Supplemental figures — Figures S1 to S8. [file jb.00400-25-s0001.pdf]

**Supplementary material**

## The *hiuABC* operon mediates xenosiderophore utilization in *Caulobacter crescentus*

Sergio Hernandez-Ortiz, Aretha Fiebig, Sean Crosson

Department of Microbiology, Genetics & Immunology, Michigan State University, East Lansing, MI, USA

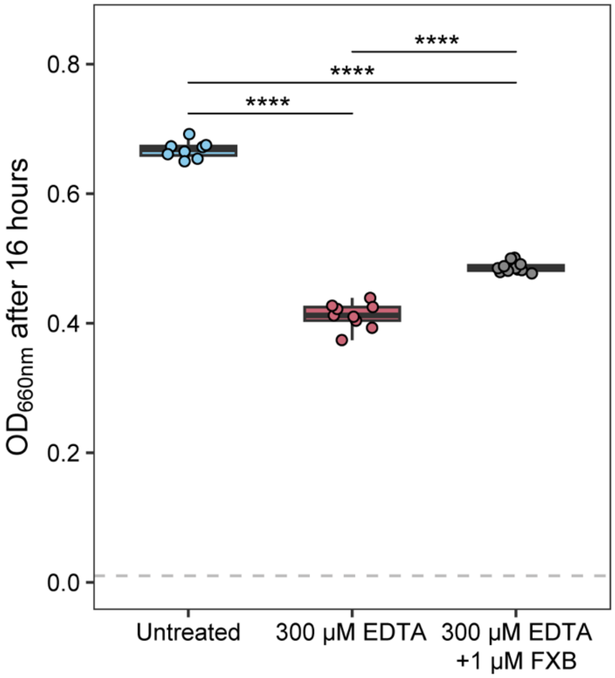


**Figure S1. Ferrioxamine B enhances the growth yield of *C. crescentus* in iron-limited PYE broth.** Optical density ($OD_{660}$) of strains after 16 hours of growth in PYE broth under the following conditions: untreated (blue), supplemented with 300 µM EDTA (pink), or with 300 µM EDTA and 1 µM ferrioxamine B (FXB). Box plots show the median and interquartile range (25th and 75th percentiles), overlaid with individual data points for each independent culture (n = 8). Statistical comparisons of $OD_{660}$ values were performed using a Kruskal–Wallis test followed by Dunn's post hoc test. Significance: ****$P < 0.0001$.

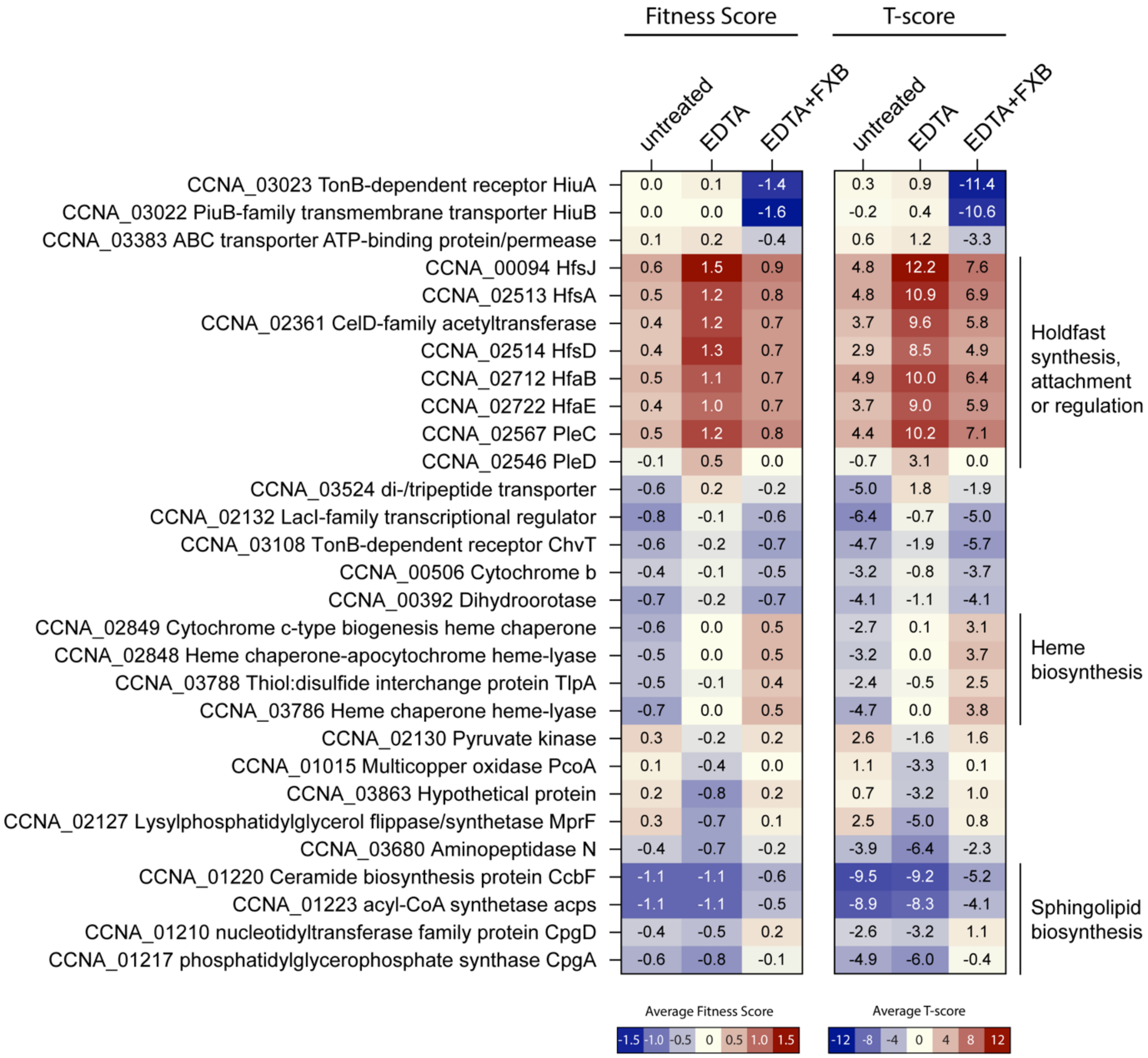


**Figure S2. Genes with high differential t-scores between EDTA and EDTA + FXB in RB-TnSeq assays.** Heatmap showing fitness scores (left panels) and corresponding t-scores (right panels) for selected genes from RB-TnSeq experiments under three conditions: untreated, 300 µM EDTA, and 300 µM EDTA + 1 µM FXB. Genes shown were selected based on exhibiting an absolute t-score difference of at least 3 between the EDTA and EDTA + FXB conditions, as defined in Figure 2 and Table S1. Each row represents a single gene, and values reflect the mean of four replicate measurements. Genes are grouped by predicted function or pathway (e.g., holdfast-dependent adhesion, heme biosynthesis, sphingolipid biosynthesis). Color scales indicate average fitness scores (left) and average t-scores (right) where positive numbers (red) indicate relative fitness enhancement of TN insertion mutants, and negative numbers (blue) indicate relative fitness defect of TN insertion mutants. EDTA = Ethylenediaminetetraacetic acid; FXB = ferrioxamine B.

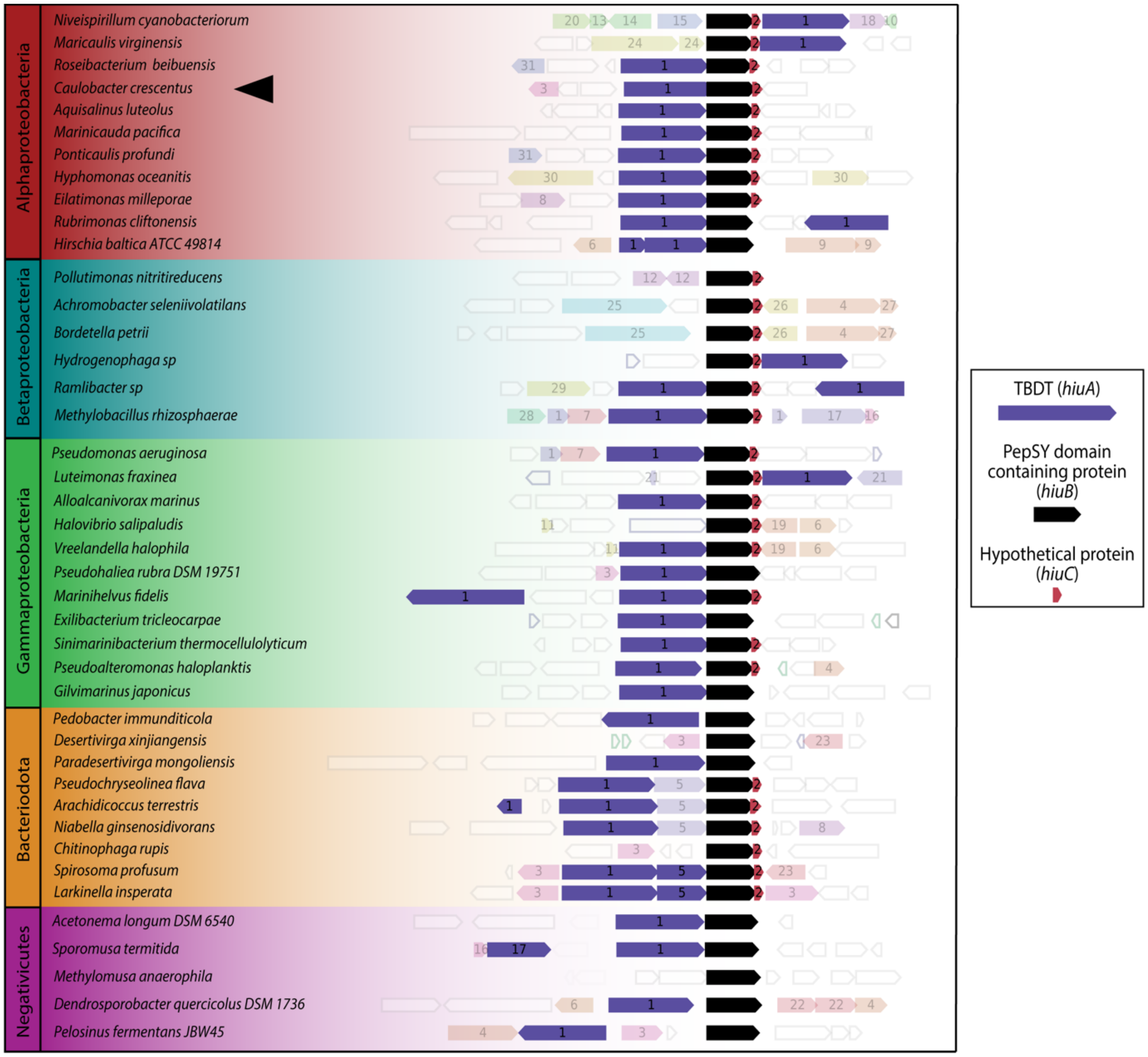


**Figure S3. Conserved *hiuABC*-like operons are found across diverse bacterial lineages.** Gene neighborhood analysis centered on *hiuB* reveals conserved synteny of *hiuA*, *hiuB*, and *hiuC* orthologs across multiple bacterial classes. Representative neighborhoods were selected based on the highest-scoring BLAST hits from members of the Proteobacteria, Bacteroidota, and Bacillota. Gene neighborhoods were visualized using webFLAGs (58). The *Caulobacter crescentus* locus is indicated by a black arrow head. Orthologs of *hiuA*, *hiuB*, and *hiuC* are colored in purple, black, and red, respectively.

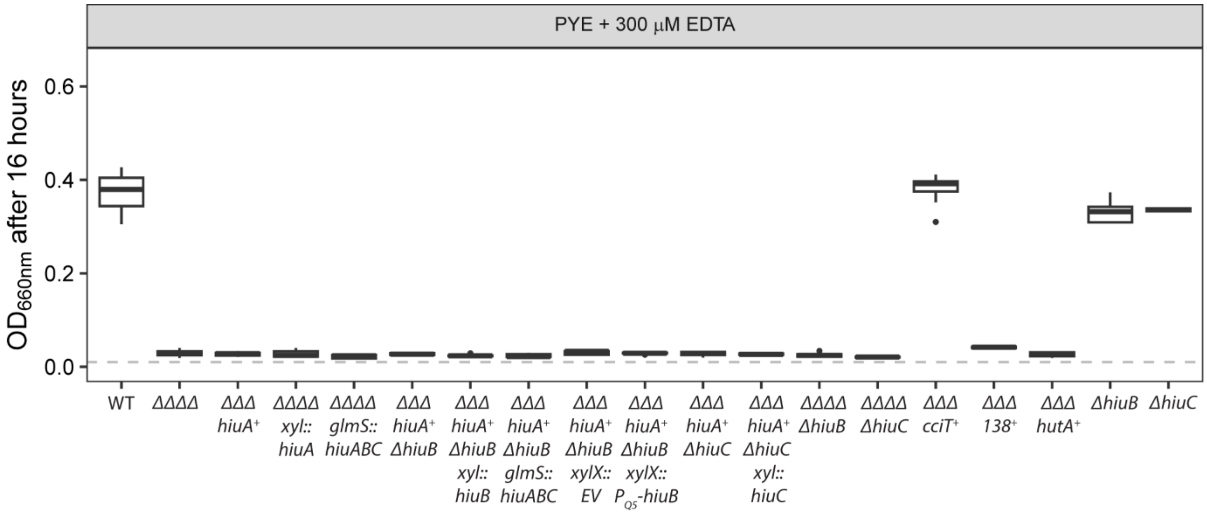


**Figure S4. Growth of all *C. crescentus* strains in the presence of 300 μM EDTA.** Optical density at 660 nm ($OD_{660}$) was measured after 16 hours of growth for all *C. crescentus* strains used in this study. Full genotypes and corresponding abbreviated genotypes for all strains are included in Table S2. Cultures were inoculated at an initial $OD_{660}$ of 0.01 (indicated by the dashed gray line) and grown in either PYE broth supplemented with 300 μM EDTA (B). Box plots display the median and interquartile range (25th to 75th percentiles) and are overlaid with individual data points from nine independent cultures per strain (n = 8, except for Δ*hiuB* and Δ*hiuC* with n=3).

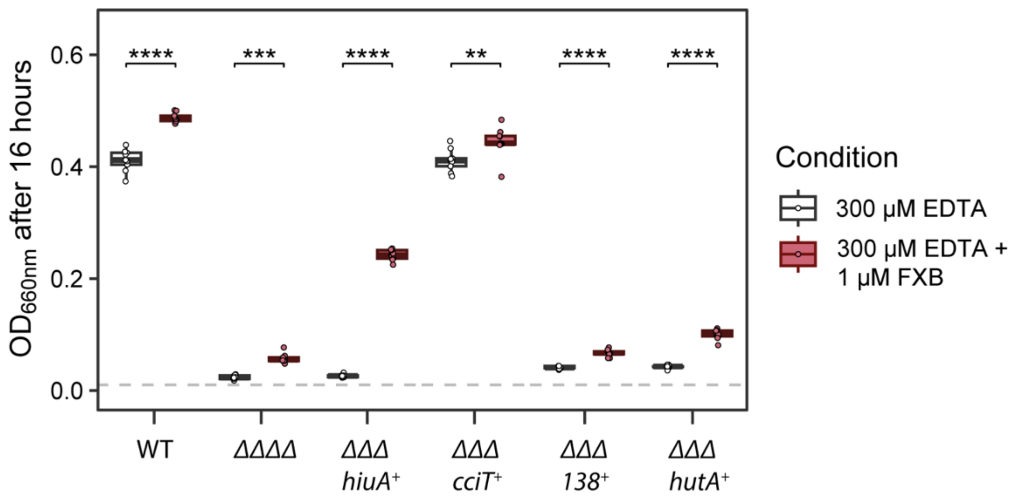


**Figure S5. *hiuA* confers optimal growth on Ferrioxamine B.** Optical density (OD) at 660 nm of cultures growth 16 hours in PYE broth treated with 300 μM EDTA and supplemented with 1 μM Ferrioxamine B (FXB). Strains include wild-type (WT), a strain lacking all four Fur-regulated TBDTs *(ΔΔΔΔ*), and strains encoding only one of each of the four Fur-regulated TBDTs (*cciT, CCNA_00138, hutA, or hiuA*) and lacking the other three (*ΔΔΔ*) Fur-regulated TBDTs. Strains were inoculated at 0.01 OD represented by the dashed gray line. Box plots reflecting the median and the 25th and 75th percentiles are overlaid with the individual data points for each independent culture (n = 9). Comparison of the OD of a strain between conditions was done through an unpaired t-test (Wilcoxon rank-sum test for *cciT*$^{+}$). Statistical significance is indicated as follows: **P < 0.01, ***P < 0.001, ****P < 0.0001.

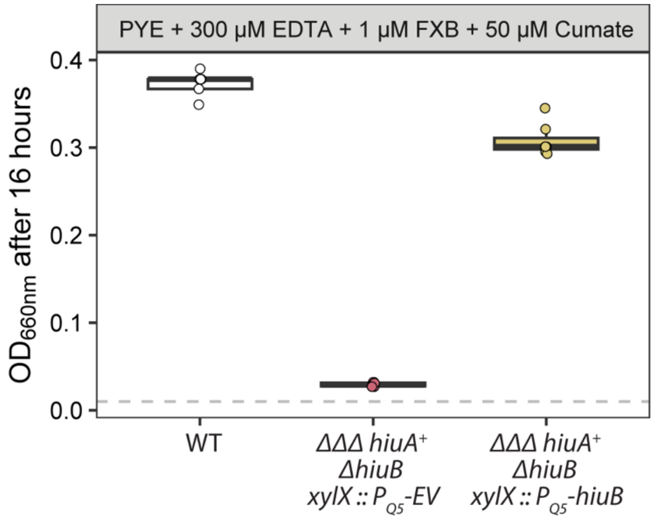


**Figure S6. *hiuB* expressed from a cumate inducible promoter complements *ΔΔΔ hiuA*$^{+}$ *ΔhiuB* strain.** Optical density ($OD_{660}$) of cultures grown in PYE with 300 µM EDTA + 1 µM Ferrioxamine E (FXE) and 50 µM cumate broth for 16 hours. Strains are wild-type (WT), and *ΔΔΔ hiuA*$^{+}$ *ΔhiuB* carrying pPTM057 empty vector (EV) or a *hiuB* insert. Strains were inoculated at 0.01 OD660 represented by the dashed gray line. Box plots reflecting the median and the 25th and 75th percentiles are overlaid with the individual data points for each independent culture (n =7).

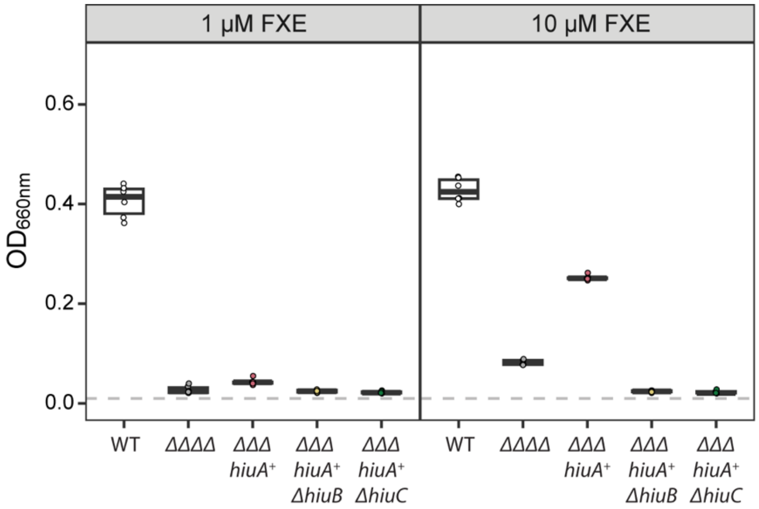


**Figure S7. The *hiuABC* operon supports growth on Ferrioxamine E**. Optical density ($OD_{660}$) of cultures grown in PYE with 300 μM EDTA and 1 μM Ferrioxamine E (FXE) or 10 μM FXE broth for 16 hours. Strains are wild-type (WT), the ΔΔΔΔ strain (lacking all four fur-regulated TonB-dependent transporters, including *hiuA*), *hiuA* $^{+}$ΔΔΔ (encoding only *hiuA*), strains lacking either *hiuB,* or *hiuC* in the *hiuA* $^{+}$*ΔΔΔ* background. Strains were inoculated at 0.01 OD660 represented by the dashed gray line. Box plots reflecting the median and the 25th and 75th percentiles are overlaid with the individual data points for each independent culture (n =9).

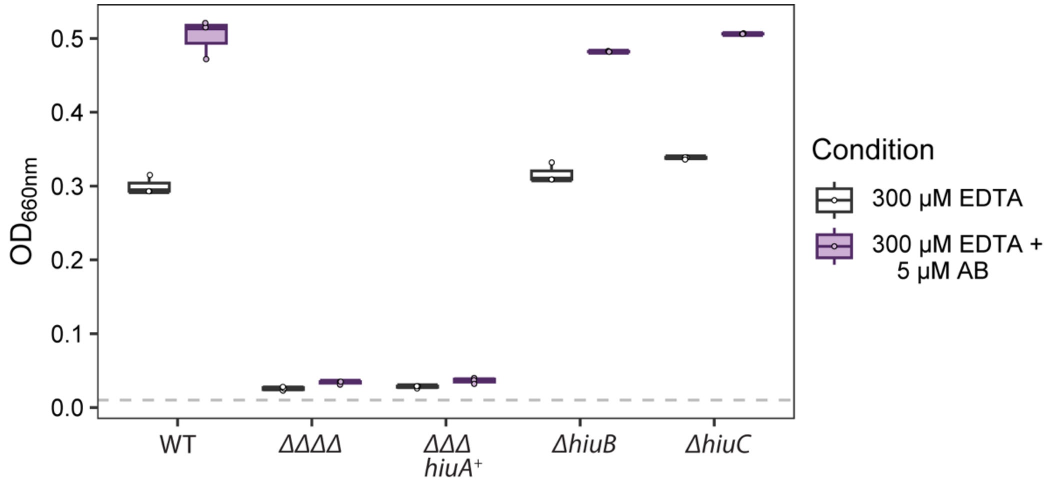


**Figure S8. *C. crescentus* aerobactin utilization does not require *hiuABC*.** Optical density ($OD_{660}$) of cultures grown in PYE with 300 μM EDTA with and without 5 μM ferri-aerobactin (AB) broth for 16 hours. Strains are wild-type (WT), the ΔΔΔΔ strain (lacking all four Fur-regulated TonB-dependent transporters, including *hiuA*), ΔΔΔ *hiuA*$^{+}$ (encoding only *hiuA*), and mutants lacking either *hiuB*, or *hiuC*. Strains were inoculated at 0.01 OD660 represented by the dashed gray line. Box plots reflecting the median and the 25th and 75th percentiles are overlaid with the individual data points for each independent culture (n =3).
